# Dose of Bicarbonate to Maintain Plasma pH During Maximal Ergometer Rowing and Consequence for Plasma Volume

**DOI:** 10.3389/fphys.2022.828708

**Published:** 2022-04-11

**Authors:** Henning Bay Nielsen, Stefanos Volianitis, Niels H. Secher

**Affiliations:** ^1^ Department of Anaesthesia and Intensive Care, Zealand University Hospital Roskilde, Institute for Clinical Medicine, University of Copenhagen, Copenhagen, Denmark; ^2^ Department of Nutrition, Exercise and Sports, Faculty of Science, University of Copenhagen, Copenhagen, Denmark; ^3^ Department of Physical Education, College of Education, Qatar University, Doha, Qatar; ^4^ Department of Anaesthesia, Rigshospitalet, Institute for Clinical Medicine, University of Copenhagen, Copenhagen, Denmark

**Keywords:** plasma volume, bicarbonate supplementation, rowing, hypoxaemia, desaturation, acidosis, bohr effect

## Abstract

Rowing performance may be enhanced by attenuated metabolic acidosis following bicarbonate (BIC) supplementation. This study evaluated the dose of BIC needed to eliminate the decrease in plasma pH during maximal ergometer rowing and assessed the consequence for change in plasma volume. Six oarsmen performed “2,000-m” maximal ergometer rowing trials with BIC (1 M; 100–325 ml) and control (CON; the same volume of isotonic saline). During CON, pH decreased from 7.42 ± 0.01 to 7.17 ± 0.04 (mean and SD; *p* < 0.05), while during BIC, pH was maintained until the sixth minute where it dropped to 7.32 ± 0.08 and was thus higher than during CON (*p* < 0.05). The buffering effect of BIC on metabolic acidosis was dose dependent and 300–325 mmol required to maintain plasma pH. Compared to CON, BIC increased plasma sodium by 4 mmol/L, bicarbonate was maintained, and lactate increased to 25 ± 7 vs. 18 ± 3 mmol/L (*p* < 0.05). Plasma volume was estimated to decrease by 24 ± 4% in CON, while with BIC the estimate was by only 7 ± 6% (*p* < 0.05) and yet BIC had no significant effect on performance [median 6 min 27 s (range 6 min 09 s to 6 min 57 s) vs. 6 min 33 s (6 min 14 s to 6 min 55 s)]. Bicarbonate administration attenuates acidosis during maximal rowing in a dose-dependent manner and the reduction in plasma volume is attenuated with little consequence for performance.

## Introduction

Bicarbonate supplementation is considered an ergogenic agent through enhanced blood buffer capacity whereby fatigue may be attenuated. Although data are inconsistent (Christensen et al., 2014; Krustrup et al., 2015) probably related to different study protocols (Maughan et al., 2018), it seems that bicarbonate administration is associated with enhanced exercise capacity (Nielsen et al., 2002a). Thus, based on a meta-analysis of moderate to high quality, it is concluded that sodium bicarbonate supplementation enhances aerobic power, anaerobic capacity, and thus performance in endurance events lasting ∼45 s to 8 min, muscle endurance, 2,000-m ergometer rowing performance, and high-intensity intermittent running (Grgic et al., 2021).

The enhanced blood buffer capacity following administration of sodium bicarbonate supports arterial O_2_ saturation (SaO_2_) by a Bohr effect on the oxygen-haemoglobin dissociation curve (Nielsen et al., 2002b) and could explain the increase in performance. However, neither pulmonary O_2_ uptake nor muscle oxygenation is affected by expanded blood buffer capacity (Nielsen et al., 2002a). The increase in blood bicarbonate expands the ability to absorb excess intramuscular hydrogen ions whereby lactate transport to blood is facilitated to serve the brain and attenuate central fatigue and thereby enhance performance (van Hall et al., 2009; Volianitis et al., 2011; Siebenmann et al., 2021). An often overlooked effect of sodium bicarbonate is that blood sodium increases. Normally exercise is associated with drop in plasma volume and that is likely attenuated by sodium bicarbonate.

The present report evaluated data from pilot studies carried out to establish the dose of bicarbonate that is required to maintain pH and SaO_2_ during maximal ergometer rowing (Nielsen et al., 2002b). As the pilot studies used different doses of bicarbonate, we evaluated whether bicarbonate influences pH in a dose-response manner. The volume administered in control and intervention settings was similar which allowed for evaluation of whether the hypertonic sodium bicarbonate solution influences an estimate of changes in plasma volume.

## Materials and Methods

Seven competitive oarsmen (age 22 ± 2 yrs, height 182 ± 3 cm, weight 78 ± 2 kg; mean with SD) participated in the study after informed consent as approved by the Ethics Committee of Copenhagen (KF 01-280/98; Nielsen et al., 2002a). On the first trial day, the rowers were asked to report their personal record for “2,000-m” ergometer rowing [median 6 min 33 s (range 6 min 03 s to 7 min 02 s)]. No subject had any disease or injury in the 3 weeks prior the trials and was not taking any medication. The subjects were fasting on the day of the experiments which took place in the morning.

Rowing was performed on an ergometer (model C; Concept II, Morrisville, VT). First, the subjects rowed for 12 min at work rates increasing from 150 to 250 W in steps of 50 W every third minute (warm-up; Nielsen et al., 2002b). Then they rowed for 5 min at an individually determined pace including several strokes at maximal intensity. After 5 min of recovery, a 2,000-m all-out time trial simulated an on-water competition. The study aimed to identify the dose of bicarbonate that would abolish acidosis during maximal rowing. Thus, the subjects received doses of sodium bicarbonate (1 M) ranging from 100—325 ml (Table 1) separated by at least 7 days. In the control setting isotonic saline, in similar volume to that provided in the bicarbonate trials, was administered. Sodium bicarbonate comes as 1 mmol/ml; a dose of, e.g., 300 mmol is therefore interchangeable to administration of 300 ml.

**TABLE 1:** Bicarbonate dose and availability of blood samples.

Subject	Saline Trial	Bicarbonate Dose (ml)			Bicarbonate Dose Used in Tables
1	−	100*	270*	300*	
2	*	200*	300		200
3	*	200	325*		325
4	*	240*			240
5	*	300*			300
6	*	325*			325
7	*	100*			100

*blood sample available.

A catheter (1.0 mm, 20 gauge) was placed in radial artery of the non-dominant arm and allowed for blood sampling during rowing. Infusion of sodium bicarbonate or saline was administered through a central catheter (1.7 mm, 16 gauge) inserted in an upper arm vein. The intended dose of sodium bicarbonate or saline to be infused was in 60-ml syringes emptied at a constant rate (app. 50–60 ml/min) according to the expected race time as reported by the rower.

**FIGURE 1:**
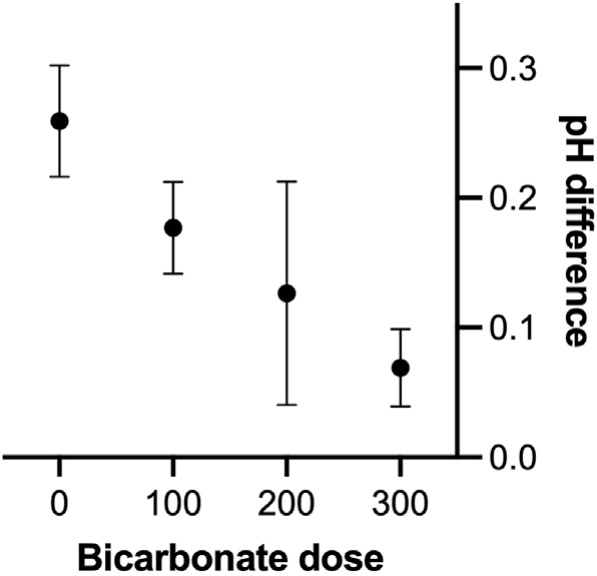
pH effect of different doses of bicarbonate administered intravenosly during maximal ergometer rowing in seven oarsmen. *X*-axis is the dose group of bicarbonate used (100: use of 100 mmol in two subjects, 200 use of 200–240 mmol in four subjects and 300 administration of 300–340 mmol in four subjects) while “0” represent the control saline trial in six subjects. *Y*-axis is the difference between the pH at rest in samples obtained in the sixth minute of rowing. It is a limitation not all subjects received same amount of bicarbonate.

Arterial blood samples were obtained anaerobically in heparinized syringes (4042E, SIMS, Radiometer, Copenhagen, Denmark) at rest, during the maximal row, and in the recovery. Samples were turned and kept on ice until analysed for blood-gas variables, haemoglobin (Hgb), haematocrit (Hct), glucose, sodium, calcium, potassium, glucose, and lactate by a ABL 615 apparatus (Radiometer) with co-oximetry for determination of haemoblobin O_2_ saturation (SaO_2_). Paired blood sample data were available for six rowers as in one subject (#1) blood sampling failed during the control trial (Table 1). Only data obtained from trials with a maximal dose of bicarbonate used for each individual went into analysis of the data as presented in Tables 2, 3. Several doses of bicarbonate were used which allowed for construction of dose-response-like plot visualising the effect of bicarbonate on pH during ergometer rowing (Figure 1).

**TABLE 2:** Blood variables during and after maximal ergometer rowing with infusion of isotonic saline.

	Rowing			Recovery		
	Rest	2 (min)	4 (min)	6 (min)	2 (min)	4 (min)
pH	7.42 ± 0.01	7.34 ± 0.01^*^	7.24 ± 0.02^*^	7.17 ± 0.04^*^	7.10 ± 0.04^*^	7.10 ± 0.03^*^
PaO_2_(kPa)	13.51 ± 1.54	11.04 ± 0.45^*^	10.81 ± 0.41^*^	10.38 ± 0.67^*^	15.97 ± 0.62^*^	16.79 ± 0.18^*^
SaO_2_ (%)	97.2 ± 0.5	95.0 ± 0.5^*^	95.0 ± 0.9^*^	90.2 ± 0.9^*^	95.3 ± 0.6^*^	95.7 ± 0.4^*^
PaCO_2_(kPa)	5.21 ± 0.42	4.61 ± 0.22	4.41 ± 0.31^*^	4.42 ± 0.18^*^	3.74 ± 0.45^*^	3.50 ± 0.47^*^
Hct (%)	43.6 ± 1.6	47.2 ± 1.6^*^	47.1 ± 2.9^*^	49.9 ± 1.7^*^	46.3 ± 2.5^*^	45.3 ± 3.4
Hgb (mM)	8.6 ± 0.4	9.6 ± 0.3^*^	9.7 ± 0.6^*^	10.1 ± 0.4^*^	9.4 ± 0.5	9.2 ± 0.7
K^+^ (mM)	3.7 ± 0.1	5.8 ± 0.4^*^	5.7 ± 0.6^*^	6.4 ± 0.6^*^	3.5 ± 0.2	3.0 ± 0.2^*^
Ca^+^ (mM)	1.18 ± 0.05	1.24 ± 0.03	1.27 ± 0.03	1.36 ± 0.03^*^	1.28 ± 0.07^*^	1.25 ± 0.09
Na^+^ (mM)	139.8 ± 1.6	145.2 ± 1.3^*^	147.0 ± 1.5^*^	147.8 ± 1.5^*^	144.3 ± 1.4^*^	143.0 ± 1.5^*^
HCO_3_(mM)	25.2 ± 1.4	18.0 ± 0.6^*^	13.9 ± 0.6^*^	11.2 ± 0.8^*^	8.4 ± 1.2^*^	7.9 ± 1.5^*^
Lactate (mM)	0.9 ± 0.4	8.5 ± 1.4^*^	13.6 ± 2.1^*^	18.3 ± 3.2^*^	17.3 ± 1.9^*^	16.5 ± 2.0^*^
Glucose (mM)	5.97 ± 0.9	5.60 ± 0.8	5.35 ± 0.5	5.90 ± 0.5	9.35 ± 1.0^*^	9.05 ± 1.3^*^

Values are arterial O_2_ pressure (PaO_2_), CO_2_ pressure (PaCO_2_), haemoglobin O_2_ saturation (SaO_2_), calcium (Ca+), haemoglobin (Hgb), haemotocrit (Hct), bicarbonate (HCO_3_^-^), bicarbonate (HCO_3_^-^), potassium (K^+^), and sodium (Na^+^) prior rowing (Rest) and in response to a 2,000-m ergometer maximal row with samples obtained after 2, 4, and 6 min as well as two and 4 mins into the recovery (*n* = 6). * different from rest, *p* < 0.05.

**TABLE 3:** Blood variables during and after maximal ergometer rowing with infusion of bicarbonate.

	Rowing			Recovery		
	Rest	2 (min)	4 (min)	6 (min)	2 (min)	4 (min)
pH	7.42 ± 0.01	7.39 ± 0.05^†^	7.36 ± 0.06^†^	7.32 ± 0.08^*†^	7.26 ± 0.09^*†^	7.24 ± 0.10^*†^
PaO_2_(kPa)	13.2 ± 1.4	11.4 ± 1.1	10.3 ± 0.6^*^	9.9 ± 0.7^*^	16.5 ± 1.0^*^	16.4 ± 1.0^*^
SaO_2_ (%)	96.7 ± 0.7	95.9 ± 0.7^*^	94.2 ± 0.1^*†^	93.2 ± 1.4^*†^	96.9 ± 0.9^†^	96.7 ± 1.1
PaCO_2_(kPa)	5.12 ± 0.64	4.95 ± 0.44	5.08 ± 0.37	5.25 ± 0.56^†^	4.17 ± 0.42	4.13 ± 0.58^†^
Hct (%)	43.7 ± 2.3	45.6 ± 3.3^*^	45.5 ± 2.5	45.4 ± 2.3^†^	43.2 ± 2.3	42.7 ± 2.3
Hgb (mM)	8.8 ± 0.5	9.22 ± 0.7^*^	9.18 ± 0.5	9.22 ± 0.5^*†^	8.5 ± 0.6	8.4 ± 0.6
K^+^ (mM)	3.7 ± 0.6	5.8 ± 0.6^*^	6.0 ± 0.4^*^	6.5 ± 0.8^*^	3.5 ± 0.4	2.9 ± 0.3^*^
Ca^+^ (mM)	1.18 ± 0.04	1.13 ± 0.03^†^	1.11 ± 0.05^†^	1.11 ± 0.07^†^	1.06 ± 0.04^*†^	1.06 ± 0.06^*†^
Na^+^ (mM)	140.5 ± 1.4	147.5 ± 1.9^*†^	149.7 ± 2.0^*†^	152.2 ± 2.9^*†^	148.0 ± 2.2^*†^	147.0 ± 2.7^*†^
HCO_3_^−^(mM)	24.5 ± 2.7	22.5 ± 3.9^†^	21.6 ± 4.2^†^	20.4 ± 4.8^*†^	14.2 ± 4.0^*†^	13.3 ± 3.9^*†^
Lactate (mM)	0.8 ± 0.2	11.2 ± 2.0^*†^	17.2 ± 6.3^*^	24.7 ± 6.7^*†^	23.5 ± 6.8^*^	22.8 ± 7.0^*^
Glucose (mM)	5.22 ± 0.5	5.28 ± 0.6	5.40 ± 0.4	5.83 ± 0.5	8.72 ± 1.1^*^	8.70 ± 1.1^*^

Values are arterial O_2_ pressure (PaO_2_), CO_2_ pressure (PaCO_2_), haemoglobin O_2_ saturation (SaO_2_), calcium (Ca^+^), haemoglobin (Hgb), haemotocrit (Hct), bicarbonate (HCO_3_^−^), potassium (K+), and sodium (Na+) prior rowing (Rest) and in response to a 2,000-m ergometer maximal row with samples after 2, 4, and 6 min as well as two and 4 mins into the recovery (*n* = 6). * different from rest, † different from respective parameter in same column and rowing as in first part of Table 2; *p* < 0.05.

**FIGURE 2:**
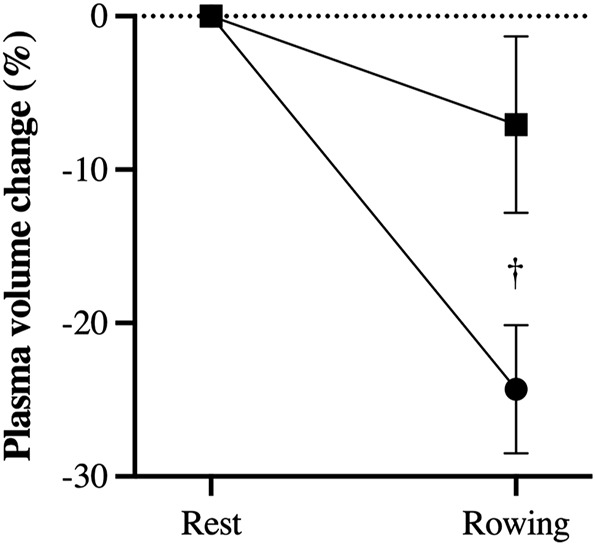
Estimated rowing induced decrease in plasma volume (%) in trials with administration of bicarbonate (square) compared to control (circle). †, difference between rowing with bicarbonate and control; *p* < 0.05.

Changes in plasma volume were estimated by modified Strauss formula (Strauss et al., 1951) as also reported by Fudim and Miller (2018) in which values for Hgb and Hct variables are incorporated. Thus, changes in plasma volume was taken as {l00 X [(Hgb B/Hgb D) X (1-Hct D)/(l- Hct B)]−100}, where B is before rowing and D during rowing.

Data are presented as mean and standard deviation (SD). For the evaluation of data in Table 2 and 3 one-way analysis of variance (ANOVA) was applied across the measure points for each parameter to be evaluated in each type of intervention. If significant interactions were found, a two-way *t*-test for paired data was used to locate differences. Evaluation of performance and plasma volume was by *t*-test only and a *p*–value < 0.05 was considered statistically significant.

## Results

The time for rowing was similar in trials with sodium bicarbonate and infusion of saline [median 6 min 27 s (range 6 min 09 s to 6 min 57 s) vs. 6 min 33 s (6 min 14 s to 6 min 55 s), respectively; *p* > 0.05] as four rowers improved their race time while two subjects (dose only 100 and 240 ml bicarbonate) demonstrated slower race time by 1 and 3 s. The perceived exertion (Borg scale) was similar in the two trials [median of 19 (range 16–19) vs. 19 (17–19), respectively; *p* > 0.05].

### Lactate, pH, and Bicarbonate

During control maximal rowing, arterial lactate increased to the last minute of exercise (Table 2) with higher values with bicarbonate (Table 3). The level of lactate remained high in the recovery and for the sodium bicarbonate trial, lactate tended to remain higher than in response to saline (*p* = 0.052 and *p* = 0.061). Thus, blood lactate increased more (by 6.6 ± 4.1 mM) in the sodium bicarbonate compared with the control trial. The maximal lactate level was 30.8 mM.

Blood bicarbonate was markedly reduced during maximal rowing with saline to a minimum of 10.2 mM and it was further reduced in the recovery (Table 2). With the infusion of sodium bicarbonate (Table 3), blood bicarbonate remained stable until the sixth min of exercise. Both during rowing and in the recovery, blood bicarbonate remained higher than in the control trial.

In response to rowing with saline, pH decreased to reach the lowest level in the sixth minute (minimum pH 7.13) and it was further reduced in the recovery (minimum pH 7.06, Table 2). With infusion of sodium bicarbonate, pH remained almost stable until the sixth minute and remained higher than in the control trial in the recovery (Table 3). Infusion of bicarbonate abolished the rowing-induced acidosis in a dose-dependent manner (Figure 1). Thus to limit the rowing-induced reduction in pH, the effective dose of bicarbonate was 300–325 mM.

### Blood-Gas Variables

In the control trial PaO_2_ decreased during maximal exercise (lowest value 9.82 kPa) and also SaO_2_ decreased to reach a minimum of 88.7% in the last minute but was re-established in the recovery (Table 2) and sodium bicarbonate trial improved SaO_2_. The saline trial reduced PaCO_2_ while rowing with sodium bicarbonate did not affcet PaCO_2_ and it remained higher than in the control trial. The Hct and Hgb increased in response to maximal rowing but with bicarbonate these variables were lower than in the control trial.

Plasma glucose was unchanged during rowing but increased in recovery without an effect of bicarbonate supplementation (Tables 2). Also potassium increased during rowing with no significant effect of sodium bicarbonate, while modest hypokalimea manifested in the recovery. During rowing with saline plasma calcium increased, while during rowing with bicarbonate it remained close to the resting level and below that observed during control exercise. Following rowing with bicarbonate, modest reduction in Ca^++^ was noted. In response to maximal rowing plasma sodium increased in both trials but to a larger extent in the trial with bicarbonate administration (by ≈ 4 mmol/L).

During rowing with saline the estimated plasma volume was reduced by 24 ± 4%, while with bicarbonate administration that reduction was by only 7 ± 6% (*p* < 0.05).

## Discussion

This study addresses two important issues 1) the effect of bicarbonate on acidosis and associated blood buffering capacity and 2) potential influence of bicarbonate on plasma volume. The data were collected retrospectively from a study that was conducted in a prospective manner. Here the effect of administration of bicarbonate i.v. (rather than orally as in most studies) is addressed.

The influence of maximal ergometer rowing on blood buffer capacity is pronounced (Nielsen et al., 1999). Evaluation of the dose-response effect of bicarbonate on pH reveals that about 300 mmol is required to eliminate acidosis during rowing, while administration of 100 mmol produces only a marginal effect. Thus, with production of lactate lowering blood pH towards, e.g., 7.1, administration of limited volume of sodium bicarbonate pH becomes only partially reversed. Importantly, intracellular pH is also affected by administration of bicarbonate (Nielsen et al., 2002b) and in perspective these observations provide an albeit indirect estimate of the anaerobic contribution to the work performed (Volianitis et al., 2020). With regards to the ergogenic effect of bicarbonate administration it is acknowledged that the intervention depends on the extend of acidosis provoked during the rowing trial (i.e., if the deviation from resting pH is marginal, enhancement of the blood buffering capacity will have minimal ergogenic effect). An important limitation is that it requires the subjects to be equally motivated in all trials as here supported by rate of perceived exertion of about 19. As indicated the data were obtained to evaluate the dose of bicarbonate needed to eliminate the decrease in pH associated with maximal rowing. The dose-response curve represented by Figure 1 was constructed only with a minimal number of observations needed to conduct the main study. The ideal dose-response curve would include data from a set-up where all subjects received different doses at separate occasions but such endeavour should be undertaken in future studies. Yet, the current data provide for a perspective on anaerobic metabolism during maximal exercise.

The other important observation relates to plasma volume changes during rowing. Exercise induces a rapid fluid-shift with a drop in plasma volume during even short-term maximal exercise (Kaltreider and Mieneely, 1940; Sullivan et al., 1993). Haemoconcentration is important for maintained arterial oxygen content and may compensate for (Schierbauer et al., 2021) or likely limit an increase in cardiac output (González-Alonso et al., 2006). By use of indirect measures to estimate changes in plasma volume, administration of sodium bicarbonate was associated with attenuated decrease in plasma volume. Likely, an increase in plasma sodium counteracts transport of fluid from the intravascular compartment. Therefore, studies evaluating the effect of sodium bicarbonate on performance should account also for plasma volume changes. Such consideration may be of relevance especially for prolonged exercise and exercise in the heat.

Blood variables evaluate the influence of rowing on blood oxygenation. As reported by Nielsen et al. (2002a), maximal ergometer rowing is associated with significant hypoxaemia as also observed for running (Rowell et al., 1964; Dempsey et al., 1984; Dempsey and Wagner, 1999). The O_2_ dissociation curve is right shifted by a decrease in pH and even a modest drop in PaO_2_ becomes of consequence for SaO_2_ that may reach 85–87% when large muscle mass is engaged (Rasmussen et al., 1991) including ergometer rowing (Hanel et al., 1994). Thus, when exercise-indued hypoxaemia is reversed by breathing an O_2_ enriched atmosphere, exercise capacity increases (Nielsen et al., 1998; Nielsen et al., 1999). This study evaluated the amount of bicarbonate needed to maintain SaO_2_ despite the drop in PaO_2_ during maximal ergometer rowing.

Interpretation of data is limited by the small number of subjects included of potential consequence for statistical significance for difference in performance and, unfortunately, blood sampling failed for one subject. Thus, an increase in performance would not be expected for those subject who received smallest dose of bicarbonate. Also, plasma volume change was based on indirect measures. The use of Hgb and Hct to estimate changes in plasma volume was proposed by Strauss et al. (1951) and Dill and Costill (1974) found the approach feasible for estimation of changes during exercise. Yet, Fudim and Miller (2018) report that in heart failure patients plasma volume calculated by formulae using Hgb/Hct correlate only moderately to a direct evaluation. Furthermore, it is likely that use of isotonic saline in the control setting has supported plasma volume. Yet, a strength of the study is that subjects acted as their own control.

We conclude that the effect of bicarbonate on arterial pH and thus oxygen saturation is dose-dependent and that a potential effect of bicarbonate on performance should take into account the effect on plasma sodium and attenuated reduction in plasma volume during maximal exercise.

## Data Availability

The original contributions presented in the study are included in the article/Supplementary Material, further inquiries can be directed to the corresponding author.
